# Shared Lifestyle-Related Risk Factors of Cardiovascular Disease and Cancer: Evidence for Joint Prevention

**DOI:** 10.1155/2023/2404806

**Published:** 2023-07-22

**Authors:** Farzad Masoudkabir, Noushin Mohammadifard, Arya Mani, Andrew Ignaszewski, Margot K. Davis, Golnaz Vaseghi, Marjan Mansourian, Christopher Franco, Carolyn Gotay, Nizal Sarrafzadegan

**Affiliations:** ^1^Cardiac Primary Prevention Research Center, Cardiovascular Diseases Research Institute, Tehran University of Medical Sciences, Tehran, Iran; ^2^Hypertension Research Center, Cardiovascular Research Institute, Isfahan University of Medical Sciences, Isfahan, Iran; ^3^Yale Cardiovascular Genetics Program, Yale Cardiovascular Research Center, Department of Internal Medicine, Yale University School of Medicine, New Haven, CT, USA; ^4^Division of Cardiology, Faculty of Medicine, University of British Columbia, Vancouver, British Columbia, Canada; ^5^Applied Physiology Research Center, Cardiovascular Research Institute, Isfahan University of Medical Sciences, Isfahan, Iran; ^6^Epidemiology and Biostatistics Department, School of Health, Isfahan University of Medical Sciences, Isfahan, Iran; ^7^School of Population & Public Health, The University of British Columbia, Vancouver, BC, Canada; ^8^Isfahan Cardiovascular Research Center, Cardiovascular Research Institute, Isfahan University of Medical Sciences, Isfahan, Iran

## Abstract

Cardiovascular disease (CVD) and cancer are leading causes of mortality and morbidity worldwide and are the major focus of the World Health Organization's joint prevention programs. While, diverse diseases, CVD and cancer, have many similarities. These include common lifestyle-related risk factors and shared environmental, metabolic, cellular, inflammatory, and genetic pathways. In this review, we will discuss the shared lifestyle-related and environmental risk factors central to both diseases and how the strategies commonly used to prevent atherosclerotic vascular disease can be applied to cancer prevention.

## 1. Introduction

Cardiovascular disease (CVD) and cancer are the leading causes of mortality and morbidity with increasing trends worldwide, and they are accountable for two-thirds of global noncommunicable disease (NCD) deaths in 2012 [1].

The 2011 United Nations High-Level Meeting on NCDs [2] stated that the global reduction of four common modifiable risk factors, including tobacco use, unhealthy diet, sedentary lifestyle, and excess alcohol consumption, can help prevent the prevalent NCDs (CVD, cancer, type 2 diabetes mellitus (T2DM), and chronic obstructive pulmonary disease).

The common modifiable risk factors for CVD and cancer are not only proper targets for joint prevention of CVD and cancer but also reflect in our emerging understanding that they link CVD and cancer through shared metabolic, genetics, and molecular mechanisms that are central to the pathogenesis of both diseases [3]. Thus, mounting evidence supports the use of medications such as aspirin, statins, inhibitors of the renin-angiotensin-aldosterone system (RAAS), thiazolidinediones, and metformin for the prevention of both CVD and cancer [3–5]. Joint risk factors and epidemiology of CVD and cancer progression provide an opportunity for improving shared risk factors and risk reduction through lifestyle modification and pharmacologic treatment [5–7]. The World Health Organization (WHO) recommends global targets and strategies for a 25% reduction in premature mortality and modifiable risk factors of NCDs until 2025 (Table 1). In this review, we will provide a perspective on the diverse shared behavioural, environmental, and metabolic risk factors of CVD and cancer. Furthermore, we will present different individual and public health strategies for the prevention of both diseases by controlling these risk factors.

## 2. Shared Behavioural Risk Factors and Strategies for Prevention

### 2.1. Smoking

According to the American Cancer Society and World Lung Foundation report, nearly 1 billion men and 250 million women use tobacco and this figure is increasing [8]. Rising tobacco sales in China alone has offset reductions in North America, the United Kingdom, Australia, and Brazil [8]. Notably, 80% of the world's smokers live in low- and middle-income countries [8, 9]. Tobacco use remains the leading cause of preventable morbidity and mortality. Smokers lose at least one decade of life expectancy compared to never-smokers [10]. The WHO currently estimates that each year tobacco use accounts for about 7 million deaths worldwide [9].

Tobacco smoking is a well-established risk factor for CVD incidence and mortality [11, 12]. Tobacco smoking accounts for 17% of CVD deaths worldwide [13]. Smoking has been recognized explicitly as a cause of coronary artery disease (CAD), cerebrovascular disease, peripheral artery disease, and abdominal aortic aneurysm [11]. Smoking also increases the incidence of valvular heart disease (e.g., aortic stenosis) [14], arrhythmia (e.g., atrial fibrillation) [15], systemic hypertension [16], and dyslipidemia (low levels of high-density lipoprotein cholesterol) [17]. Secondhand smoke exposure is also associated with CVD in nonsmoking adults. Never-smokers exposed to secondhand smoke at home or work increase their CAD and stroke risk by 25–30% [18]. There are a multitude of experimental, clinical, and epidemiological studies providing sufficient evidence for the causal relationship between tobacco smoking and cancer [12, 19]. Tobacco use accounts for at least 30% of all cancer deaths [20]. Tobacco smoking can cause cancer almost everywhere in the body, including cancers of the lung, oral cavity and pharynx, nasal cavities and paranasal sinuses, oesophagus, stomach, liver, pancreas, colorectal, bladder, kidney and renal pelvis, cervix, endometrium, and myeloid leukemia [12, 19]. Moreover, like CVD, never-smokers exposed to secondhand tobacco smoke at home or workplace have a 16–30% increase in the risk of developing lung cancer [19].

Despite the lack of large-scale, randomized trials concerning the CAD and stroke risk reduction associated with smoking cessation, observational studies consistently demonstrate the clear benefits of smoking cessation and approach that in never-smokers after 3–5 years for CAD and 5–15 years for stroke. Smoking cessation benefits virtually all smokers, regardless of the duration or intensity of their smoking, degree of illness, or age at quitting [11]. Smoking cessation is associated with a decreased risk of cancer. After quitting, the risk of cancer drops by half after five years for lung cancer and after two years for cancers of the oral cavity and pharynx, oesophagus, and bladder [21]. Legislations banning smoking in indoor public places and workplaces were implemented in some areas of the world and reported a 12% reduction in hospitalizations for acute coronary events. Therefore, smoking cessation can effectively reduce the risk of all-cause mortality and some cancers, such as lung mortality. It should be mentioned that sex differences have been observed after smoking cessation and risk reduction, which is stronger in females [22].

Smoking cessation is the most urgent and cost-effective priority out of the four very cost-effective interventions (including tobacco control, reducing alcohol consumption, promoting physical activity, and a healthy diet) for prevention and control of NCDs known as “WHO's best buys for prevention of NCDs” [23]. However, there are multiple barriers and challenges to tobacco control at both individual and community levels. Community-based lifestyle intervention programs have been associated with varying degrees of improvement in smoking behaviour. For example, the “Isfahan Healthy Heart Program” (IHHP) is a comprehensive, integrated community-based program for NCD prevention and healthy lifestyle promotion that consists of interventions on smoking, diet, and physical activity in Iran [24]. Multiple interventions were used, from education of the population and health care providers to environmental and policy changes. Specific interventions for high-risk individuals and CVD patients after hospital discharge were also implemented. The investigators observed that, after four years of interventions, adherence to a healthy diet significantly improved in the intervention population in contrast to the control population. However, the interventions impacted smoking cessation in men but not in women [24], which points out the need to modify interventional activities in this group. However, only 6% of those who attempted to quit succeeded [25]. One reason for the low success rate may be that few smokers use any smoking cessation treatment when attempting to quit. Evidence from randomized controlled trials has clearly identified several individual-level treatments as effective for smokers who want help quitting. These approaches include brief clinical interventions, which are presented in Table 2. However, combining these strategies is more effective for smoking cessation than using either alone [26].

In 2003, the WHO developed the Framework Convention on Tobacco Control (FCTC) as the first global treaty for tobacco control and stress reductions in both demand and supply of tobacco [27]. In 2007, the WHO offered a practical strategy to scale up the implementation of provisions of the WHO FCTC on the ground, which is summarized as “MPOWER” [9]. The 6 MPOWER measures are as follows: monitoring tobacco use and prevention policies, protecting people from tobacco use, offering help to quit tobacco use, warning about the dangers of tobacco, enforcing bans on tobacco advertising, promotion, and sponsorship, and raising taxes on tobacco. Full implementation of FCTC strategies would save more than 5 million deaths in 23 large low- and middle-income countries alone during a 10-year period [23]. Available evidence from the same analysis indicates that implementing all these interventions would cost less than 0.40$ per person per year in low-income and lower-middle-income countries and 0.5–1.0$ per person per year in upper-middle-income countries [19]. Other than cigarettes, tobacco has other forms, such as cigars, pipes, and hookah (water pipe or shisha). Hookah smoking is a traditional method of tobacco smoking that has high popularity in both developing and developed countries as it is misperceived as less hazardous than cigarettes [28]. It has been shown that hookah smoke contains a wide range of toxic substances. Current evidence indicates that hookah is associated with adverse effects on the cardiovascular system as it increases the systolic and diastolic blood pressure by 12–16 mmHg and 2–8 mmHg, respectively, leading to a rise in the heart rate by 6–15 beats per minute. In addition, it is associated with a reduction in baroreflex sensitivity, which is a risk factor for coronary artery diseases. Furthermore, hookah is associated with lung and nasopharyngeal cancer as well as oral squamous cell carcinoma. However, the existing data are conflicting because research on the health effects of hookah still harbours a lot of deficiencies, and there is a lack of well-defined studies in the field. The current studies suffer from important design and content issues that need to be considered for future cessation trials in hookah smokers. Cigar smoking has also been shown to double the risk of upper aerodigestive tract and lung cancers and enhance the CAD risk by just below 30% [29]. While current cigarette smokers had higher levels of various inflammatory markers, including C-reactive protein, white blood cells, and fibrinogen, primary pipe/cigar smokers had similar levels of these factors compared with never-smokers [30]. Importantly, however, inflammatory marker levels remained elevated among those who had switched from cigarettes to pipe/cigar (i.e., secondary pipe/cigar smokers) even more than 20 years ago. A study evaluating the combined primary and secondary pipe/cigar smokers evinced that the risk of all-cause mortality, cardiovascular mortality, and major CHD events in this group lies between never-smokers and light cigarette smokers, while their risk of lung cancer was similar to that of light cigarette smokers [31]. Although some studies have reported a lower risk of smoking-associated conditions among cigar/pipe smokers compared to their cigarette-smoking counterparts, this difference is mainly attributable to behavioural factors rather than pathophysiological causes. In other words, cigar smokers are generally younger men and have lower intensity of tobacco smoking than cigarette smokers. Furthermore, they are less inclined to inhale the smoke, thereby reducing their exposure levels [32]. Consequently, the health consequences of tobacco smoking did not differ significantly between pipe/cigar and cigarette smokers at comparable levels of tobacco consumption [33].

### 2.2. Opium Abuse

In developing countries of the Middle East region and in many Asian nations, opium is the second most commonly abused substance, after tobacco [34]. There are consistent findings [18, 34–36] demonstrating the strong association of opium consumption with both CVD and cancers (oesophagus, stomach, larynx, lung, and urinary bladder). The potential mechanisms linking opium consumption to CVD and cancer, including inflammation, plasminogen activator inhibitor-1, adiponectin, and homocysteine, have been comprehensively reviewed by our team [37]. Table 2 shows the appropriate strategies to reduce opium abuse.

### 2.3. Alcohol Consumption

About 2.8 million deaths are attributed to alcohol use worldwide, which is also the leading risk factor among individuals aged 15–49 years [38]. A causal association has been established between drinking alcohol and cancers of the oral cavity, oesophagus, pharynx, larynx, liver, colorectum, and breast [39]. However, the relationship between alcohol consumption and CAD and cerebrovascular diseases is complex. It is well demonstrated that alcohol consumption increases the risk of hypertensive disease, atrial fibrillation, and hemorrhagic stroke [1]. Moreover, there is consistent evidence that heavy or binge drinkers have increased risks of CAD, sudden cardiac death, and ischemic stroke [40]; yet, on the other hand, controversial evidence suggests that lower levels and particular patterns of alcohol consumption in some populations may reduce the risk of CAD, ischemic stroke, and associated mortality. This may be related to wide individual variability in the bioavailability of the polyphenols. Also, gut microbial metabolism may play a major role in the biological activity of many beer polyphenols [1, 40]. However, the beneficial effects of lower levels of alcohol consumption, if any, tend to disappear if the drinking patterns are characterized by heavy episodic drinking [1, 41]. Thus, the WHO has proposed at least a 10% reduction in the harmful use of alcohol as one of the global targets to be attained by 2025 to achieve the main goal of 25% reduction in premature death due to NCD [1].

The 2015 U.S. Dietary Guidelines for Americans limit moderate consumption of alcohol to 1 drink/day for women and two drinks/day for men [42]. The accumulated research findings indicate that population-based policy options such as raising the taxes on alcohol, restricting access to retail alcohol, and enforcing bans on alcohol advertising are highly cost-effective in reducing alcohol-attributable deaths and disabilities at the population level [40, 41]. Moreover, health professionals play an important role in reducing the harmful use of alcohol (Table 2).

### 2.4. Unhealthy Diet

Unhealthy diets, known as diets high in sugars, salt, saturated and trans fatty acids, and low fruit and vegetable consumption, have been consistently shown to increase the risk of CVDs and some forms of cancer [26, 43, 44]. Globally, 20% of mortality (11 million deaths) and 255 million DALYs were attributable to unhealthy diets in 2017. High sodium intake, low whole grains intake, and low fruit intake were the leading dietary risk factors for deaths and DALYs [45].

Although an unhealthy diet is linked to an increased risk of CVD and cancer, each component has a major connection with a specific type of cancer or cardiovascular disease. The strongest association between high salt intake and cancer has been reported for gastric cancer [46] and among CVDs with hypertension [47]. Various studies noted inconsistencies in the relationship between mineral intake and the risk of CVDs and cancers; however, it has been reported that a U-shaped association exists between trace minerals and cardiovascular events, cancers, their risk factors as well as all-cause mortality [48].

Those peculiar dietary habits, nowadays known as the “Mediterranean diet,” are rich in fish, fruits, vegetables, whole grains, legumes/nuts, and as a common culinary trait, the routine use of extra virgin olive oil [49]. A 2-point increase in adherence to the Mediterranean diet resulted in an 8% reduction in overall mortality, a 10% reduced risk of CVD, and a 4% reduction in neoplastic diseases [50, 51]. Dietary improvements easily rival those seen with more established means used to prevent CVDs, such as aspirin, beta-blockers, angiotensin-converting enzyme inhibitors, and exercise [49]. It has been suggested that dietary fiber can reduce the risk of mortality of both CVD and cancer [52]. For decades, dietary guidelines have focused on reducing total fat and saturated fatty acid intake, based on the presumption that replacing saturated fatty acids with carbohydrate and unsaturated fats will lower LDL cholesterol and should therefore reduce CVD events. However, there is a growing number of scientists stating that advice to restrict saturated fatty acids is largely based on magnifying some observational data, despite the existence of several randomized trials and observational studies reporting that replacing saturated fatty acids with mostly n-6 polyunsaturated fatty acids is unlikely to reduce CVD events or total mortality [53–55]. The initial results of the Prospective Urban Rural Epidemiological (PURE) study, as a large prospective observational study to assess the association of nutrients with CVD and mortality on more than 135000 individuals from 18 countries across five continents for a median of 7.4 years of follow-up, reported that fruits, legumes, and raw vegetables were significantly associated with lower mortality [56] and carbohydrate intake was associated with increased mortality, and they observed that higher intakes of fats (including saturated fatty acids, monounsaturated fatty acids, and total polyunsaturated fatty acids) were each associated with lower cardiovascular and total mortality [57]. Although PURE has its own critics [58] and some questions should be clarified, it is likely that global dietary guidelines be reconsidered in light of the consistent findings of the PURE study, with the conclusions from meta-analyses of other observational studies [53–55] and the results of recent randomized controlled trials [54]. Table 2 indicates effective strategies for promoting a healthy diet.

### 2.5. Physical Inactivity

In 2010, insufficient physical activity, defined as less than 150 minutes of moderate-intensity physical activity per week or equivalent, was observed in 23% of adults aged 18 years and over and resulted in 3.2 million deaths and 69.3 million DALYs [59]. According to findings of meta-analyses, insufficient physical activity has been associated with a 20–40% increased risk of colon cancer, postmenopausal breast cancer, and endometrial cancer [60, 61]. A more recent study provided some evidence that replacing sedentary time with physical activity significantly reduces the mortality and risk of both CVD and cancer [62]. The WHO has targeted at least a 10% reduction in the prevalence of insufficient physical activity by 2025 [1]. According to the WHO recommendations, children and youth aged 5–17 should accumulate at least 60 minutes of moderate-to-vigorous-intensity physical activity daily and adults aged 18–64 should do at least 150 minutes/week of moderate-intensity (for example, brisk walking, jogging, gardening) or 75 minutes/week of vigorous-intensity aerobic physical activity [1].

There are several potential mechanisms justifying the protective role of physical activity in CVD and cancer [63]. Chronic low-grade inflammation and oxidative stress play pivotal roles in the pathogenesis of both CVD and cancer. One of the mechanisms seems to be playing the important role of skeletal muscles in secreting anti-inflammatory cytokines, including muscle-derived interleukin 6 (IL-6), interleukin 8 (IL-8), interleukin 15 (IL-15), and interleukin 1 receptor antagonist (IL-1ra). Interestingly, unlike IL-6 produced by monocytes and macrophages that exert a proinflammatory effect, muscle-derived IL-6 acts as an anti-inflammatory cytokine. Meanwhile, regular moderate physical activity can increase the total antioxidant capacity of defense and be responsible for the elimination of reactive oxygen species (ROS), which are responsible for oxidative stress [63]. Numerous studies have shown the importance of gut microbiota in the pathogenesis of CVD, gastrointestinal tract, and other cancers, including the head, neck, breast, and prostate. There is evidence that regular physical activity induces changes in the microbial composition of the host, which could protect from CVD and cancers of the gastrointestinal tract [63, 64].

### 2.6. Sleep Disorders

Sleep, as a part of lifestyle, plays an important role in determining the quality of life and health [65]. It is now well established that obstructive sleep apnea (OSA), abnormally short or long habitual sleep, and late bedtime are associated with an increased risk of CVDs events, including stroke, CAD, hypertension, left ventricular dysfunction, arrhythmias, and sudden cardiac death [66–69]. Numerous epidemiologic studies spanning the last decade have demonstrated that altered sleep duration and OSA are associated with a higher incidence [70] or adverse prognosis [71] of several solid tumors, including colorectal, thyroid, and lung cancers. Moreover, mounting epidemiologic studies have reported that night shift working is associated with a significantly increased risk of developing a number of different malignancies, including breast [72], colorectal [73], prostate [74], and endometrial [75] cancers. Regardless of the proposed underlying mechanisms [71, 76], which are most common between CVDs and cancer, sleep disorders might be considered an emerging lifestyle-related risk factor for CVDs and cancer. Keeping good sleep could be another potential recommendation to prevent these disorders [68, 76]. A large cohort confirmed a U-shaped association between sleep duration and all-cause and CVD mortality among healthy middle-aged men and women. It demonstrated that 7-8 hours would be the best sleep duration [77]. According to the joint consensus statement of the American Academy of Sleep Medicine and Sleep Research Society on the amount of sleep, adults should sleep 7 hours or more per night on a regular basis to promote optimal health [78]. Meanwhile, sleeping more than 9 hours per night on a regular basis may be appropriate for young adults, individuals recovering from sleep debt, and individuals with illnesses [78]. Further large-scale interventional studies are needed to refine the definition of “Good Sleep” and clarify the potential role of identifying and managing sleep disorders in prevention of CVDs and cancers.

The mechanisms linking sleep disorders to CVD and cancer have been discussed in recent publications [79]. It is well known that NF-*κ*B is a key proinflammatory transcription factor that is involved in the pathogenesis of both CVD and cancer. There is evidence that sleep disturbance activates NF-*κ*B [80]. Similarly, insufficient sleep increases C-reactive protein, an important proinflammatory mediator involved in the pathogenesis of both CVD and cancer. There is a large body of evidence that melatonin not only has beneficial effects on various cardiovascular diseases [81] but may also have a protective role against the initiation, progression, and metastasis of cancer [82]. Hence, reduced melatonin production due to exposure to light at night in those with an evening chronotype or those who do shift work may be another potential mechanism justifying the increased risk of CVD and cancer.

## 3. Shared Environmental Risk Factors and Strategies of Prevention

### 3.1. Air Pollution

Ambient air pollution is a mixture of thousands of components. From a health perspective, the most important components include airborne particulate matter (PM) and the gaseous pollutants ozone (O_3_), nitrogen dioxide (NO_2_)_,_ volatile organic compounds (including benzene), carbon monoxide (C.O.), and sulfur dioxide (SO_2_). Air pollution has a wide range of hazardous effects on human health and is a major global health problem. According to the Global Burden of Disease (GBD) study 2016, 7.5% of global deaths were attributed to ambient air pollution, the sixth risk factor attributable to DALYs [83]. In 2019, two major forms of air pollution contributing to CVD burden were PMs smaller than 2.5 mm (PM 2.5) and household air pollution (HAP). CVDs were the cause of approximately 50% and 30% of PM 2.5- and HAP-attributable DALYs [84].

There are several studies in diverse populations linking short-term and long-term air pollution exposure with an increased risk of CVD and venous thrombotic events [85–88]. Moreover, a systematic review and meta-analysis of 35 studies showed a positive association between short-term increases in gaseous components and particulate matter (PM) with the risk of hospitalization and mortality in heart failure patients [89]. In 2013, the WHO's specialized cancer agency, the International Agency for Research on Cancer (IARC), announced that sufficient evidence shows that exposure to outdoor air pollution and PM, particularly PM_10_ and PM_2.5_, causes lung cancer and it is also associated with bladder cancer. Moreover, in a meta-analysis published in 2015, household air pollution (a well-established risk factor for lung cancer) was a risk factor for other types of cancers, including those of the cervix and upper aerodigestive tract (oral, nasopharyngeal, pharyngeal, and laryngeal cancers) [90]. This association is probably due to the carcinogenic properties of inhaled polycyclic aromatic hydrocarbons, a major component of household air pollution, on the mucosal and endothelial lining of the upper aerodigestive tract [90]. In addition to CVD, cancer, and respiratory diseases, multiple systematic reviews and meta-analyses have also linked diabetes to air pollutant exposure [91, 92].

On the other hand, a decrease of 10 *μ*g per cubic meter in the concentration of fine particulate matter was associated with an estimated increase of 0.6 years in life expectancy [93].

Available evidence indicates that small particulate pollution (PM_10_ and PM_2.5_) has health impacts even at very low concentrations. Indeed, there is no safe level of exposure or a threshold below which no adverse health effects occur. Therefore, the WHO 2005 guideline limits aimed to achieve the lowest concentrations of PM possible. The WHO guideline recommends the mean PM_2.5_ concentrations to be less than 10 *μ*g/m^3^ annually and less than 25 *μ*g/m^3^ in 24 hours and the mean PM_10_ concentrations to be less than 20 *μ*g/m^3^ annually and less than 50 *μ*g/m^3^ in 24 hours. There are serious risks to health not only from exposure to PM but also from exposure to ozone (O_3_), nitrogen dioxide (NO_2_), and sulfur dioxide (SO_2_). The WHO has set values for these pollutants, as shown in Table 3. The WHO Air Quality Guidelines estimate that reducing annual average particulate matter (PM_10_) concentrations from levels of 70 *μ*g/m^3^, common in many developing cities, to the WHO guideline level of 20 *μ*g/m^3^ could reduce air pollution-related deaths by around 15%. Governments should develop policies and investments supporting cleaner transport, energy-efficient housing, power generation, industry, and better municipal waste management that would reduce key sources of urban outdoor air pollution. By reducing air pollution levels, particularly PM_2.5_, PM_10_, and NO_2_, countries can reduce the burden of both CVD and cancer [94].

There are strategies to reduce the adverse effects of air pollutants (Table 2). Limited evidence suggests that respirators may be effective in some circumstances. Research on mechanisms underlying the adverse health effects of air pollution has suggested potential pharmaceutical or chemopreventive interventions, such as antioxidant or antithrombotic agents, but there is still no evidence of the health outcomes [95].

Green space may improve health by enabling physical activity and stress recovery or decreasing pollution levels. It may reduce the natural cause and mortality from respiratory and cardiovascular disease (CVD), including ischemic heart disease, stroke, and hypertension. These protective effects were stronger in younger individuals and in women. Estimates remained virtually unchanged after incremental adjustment for air pollution and transportation noise, and mediation by these environmental factors was found to be small [96]. It has been suggested that residential green spaces reduced the risk of mortality independently from other environmental exposures. This suggests that the protective effect goes beyond the absence of pollution sources [96].

### 3.2. Soil and Water Pollutants

There is increasing concern regarding the overall health effects of chronic exposure to various heavy metal pollutants in the water and soil. This is particularly true of heavy metals such as arsenic, cadmium, mercury, and lead. A number of publications indicate that heavy metals can alter cellular metabolic pathways through the induction of a pro-oxidative state [97].

A recent systematic review indicated that studies from multiple countries in populations with different ethnicities consistently found an association between chronic exposure to high levels of arsenic and CVD [98]. Arsenic-related CVDs include hypertension, coronary artery diseases, peripheral vascular disease, and severe arteriosclerosis. This pollutant also significantly affects many types of cancers. The skin and several types of internal cancers have been associated with arsenic ingestion [98]. Arsenic is found in drinking water, cigarettes, foods, and industry occupational environments [99]. Documents established that contaminated groundwater used to cultivate rice and vegetables might be an important pathway of arsenic ingestion [99].

Cadmium is a toxic, nonessential, bioaccumulating, and highly persistent metal with a variety of adverse health effects. Cadmium has been widely dispersed into the environment through phosphate fertilizers, sewage sludge, tobacco, plastics, and foods (including rice and some vegetables) [100]. Urine cadmium, a biomarker of long-term exposure to cadmium, is related to increased cardiovascular morbidity and mortality. Moreover, experimental studies support a role for cadmium in atherosclerosis, including increased endothelial permeability by inhibiting cell proliferation and promoting cell death [101]. As an estrogen-mimicking chemical, cadmium may be associated with increased susceptibility to hormone-dependent cancers, including breast, lung, pancreatic, and endometrial cancer [101].

Many scientific resources have documented that dental amalgam is the largest mercury source in most people with several amalgam fillings, which is also proven by autopsy findings [102]. This toxic metal has major effects on weakening the immune system and facilitating cancer. Furthermore, mercury inactivates catecholamine-0-methyltransferase, which results in increased serum and urinary epinephrine, norepinephrine, and dopamine. This effect can increase B.P. and may be a clinical clue to mercury toxicity [103]. Mercury toxicity has been shown to lead to hypertension, CAD, myocardial infarction, cardiac arrhythmias, sudden death, reduced heart rate variability, increased carotid intima-media thickness, cerebrovascular accident (CVA), and generalized atherosclerosis [103]. Mercury exposure adversely affects thyrocytes and can majorly affect thyroid cancer. Moreover, some studies have found an increased risk of lung, breast, hematologic, kidney, and brain cancers among dental workers.

Lead is one of the heavy metals derived from both natural and manufactured sources. The sources of this metal are lead-acid batteries, pigments, rolled extrusions, munitions, and cable sheathing. There is evidence that lead exposure could be related to an increased risk of both CVD and cancer [104, 105]. Recent experimental studies confirm an association between lead exposure and high serum levels of homocysteine [105]. Epidemiologic studies have frequently demonstrated the association of exposure to inorganic lead lung, stomach, kidney, and brain cancers [105]. More recently, an increased risk of Hodgkin's lymphoma, lung, and recatal cancers has been reported in persons who are occupationally exposed to lead [105].

The abovementioned evidence calls for urgent action to control and reduce water and soil pollution with heavy metals as a joint preventive strategy for both CVD and cancer.

## 4. Shared Metabolic Risk Factors and Strategies for Prevention

We have previously presented the role of metabolic risk factors in CVD and cancer, their process of action, and pharmacological as well as nonpharmacological methods of prevention [7]. Here, we will discuss them briefly.

### 4.1. Metabolic Syndrome

Metabolic syndrome (MetS) describes a constellation of linked metabolic abnormalities that are associated with increased risks of CVDs and type 2 diabetes mellitus (T2DM). The worldwide prevalence of MetS varies between 5 and 35% depending on gender and race, and the International Diabetes Federation (IDF) estimates that overall, one-quarter of the world's population has MetS [106]. Recently, the links between MetS and its components have also been tied to the development of cancer [107]. A number of studies have recently been published from the Metabolic Syndrome and Cancer Project (Me-Can) cohort in Austria, Sweden, and Norway, indicating a significant association between MetS and its individual components on the risk of a wide variety of human cancers [108, 109].

It is believed that insulin resistance is the pivotal pathophysiological process underlying MetS. Adenosine 5′ monophosphate-activated protein kinase (AMPK) is a key regulator of cellular metabolism and plays a critical role in maintaining glucose homeostasis and improving insulin sensitivity [110]. Emerging evidence supports protective effects of AMPK in MetS, CVD, and cancer [3]. PPAR-*γ* is a transcription factor that regulates the expression of multiple genes involved in lipid and glucose homeostasis. In addition to its effect on glucose metabolism and insulin resistance, PPAR-*γ* activation can reduce atherosclerosis, lower blood pressure, and act as a tumor suppressor by reducing proliferation and angiogenesis and promoting differentiation [3].

### 4.2. Diabetes Mellitus

T2DM is not only one of the major predisposing factors for atherosclerosis and CVDs but has also been linked to an increased risk of developing various types of human cancer in recent meta-analyses [111]. The CPS II study examined the association between T2DM and cancer mortality in 467,922 men and 588,321 women in the U.S. Data from 16-year and 26-year follow-ups showed that T2DM is associated with an increased risk of mortality from different kinds of cancer [112] and that this association is independent of the body mass index (BMI). In addition to an increase in incidence and mortality, diabetes is associated with an increase in distant metastases in breast cancer patients, as well as a greater chance of cancer recurrence in breast, lung, and colorectal cancer patients [113].

The underlying mechanisms for the linkage of T2DM, CVD, and cancer have been extensively discussed in recent publications. In addition to the abovementioned mechanism regarding AMPK and PPAR-*γ*, there is evidence that inflammation and oxidative stress, hyperglycemia, and hyperinsulinemia in diabetic patients play a major role in developing CVD and cancer [114].

### 4.3. Dyslipidemia

Dyslipidemia includes low high-density lipoprotein cholesterol (HDL), high LDL, and high serum triglycerides levels. All these types of dyslipidemia have been consistently shown to be major risk factors for atherosclerosis and CVDs. In addition to atherosclerosis, there is a growing body of evidence indicating a link between dyslipidemia and developing oesophageal, colorectal, lung, renal, and thyroid cancers [115]. It has been observed that low serum HDL levels are associated with lung, prostate, and liver cancers incidence as well as non-Hodgkin lymphoma [116]. Low serum HDL was also suggested to be a marker for the increased breast cancer risk in premenopausal and postmenopausal women since it might reflect an unfavorable hormonal profile with particularly increased estrogen levels, especially in obese women [116]. Investigators of the Me-Can cohort also reported that triglycerides are associated with an increased risk of colon, respiratory tract, kidney, and thyroid cancers and melanoma in men and respiratory, cervical, and nonmelanoma skin cancers in women [117]. Moreover, a high LDL cholesterol concentration is associated with a higher risk of hematological cancer [118] and breast cancer [119]. Possible mechanisms relating HDL and triglycerides with cancer risks are speculations that HDL exerts anti-inflammatory and antioxidant effects, and hypertriglyceridemia is associated with the development of oxidative stress and reactive oxygen species [119].

### 4.4. Hypertension

Hypertension is, without a doubt, a major risk factor for CVDs. A meta-analysis of studies evaluating the possible association of hypertension with 18 types of cancers confirmed the positive association between hypertension and the risk of kidney cancer and also found possible positive associations between hypertension and the risk of colorectal, breast, endometrial, liver, and oesophageal cancers [120]. Although the exact underlying mechanisms connecting hypertension with the increased risk of cancer remain to be clarified, the renin-angiotensin-aldosterone system (RAAS) seems to be a major connecting factor in hypertension, CVD, and cancer nexus [4]. In addition to increasing blood pressure, activation of the RAAS can promote angiogenesis [121, 122], cell proliferation [123], and DNA synthesis [124]. In animal models, blocking angiotensin-II decreases preneoplastic lesions, cell growth, angiogenesis, and VEGF levels [125]. However, evidence regarding the impact of long-term exposure to angiotensin-converting enzyme inhibitors (ACEIs) and angiotensin receptor blockers (ARBs) is controversial. While some studies suggest improved overall survival and progression-free survival of cancer patients using ACEIs/ARBs, there is evidence from trials that exposure to ARBs for more than 2.5–3 years could be associated with a combined increased risk of all cancers, particularly lung cancer [126].

### 4.5. Overweight and Obesity

Obesity is recognized as a continuously growing global public health problem, such as diabetes, hypertension, and cancer. Over the past four decades, from 1975 to 2014, the prevalence of obesity has increased from 3.2% to 10.8% in men and from 6.4% to 14.9% in women worldwide [127]. It is widely accepted that obesity increases the risk of a variety of acute and chronic disorders, such as T2DM, dyslipidemia, and CVD [6]. Furthermore, epidemiological studies have demonstrated a robust link between obesity and cancer development at numerous sites, in particular the breast (postmenopausal), endometrium, oesophagus, pancreas, colorectum, and kidney [6, 7]. Obesity also increases cancer-related mortality, and overall, 14% of all cancer deaths in men and 20% of all cancer deaths in women are attributable to overweight and obesity [128]. A recent study revealed that weight loss following bariatric surgery may lead to a reduction in mortality by 40%, reducing CAD death by and due to cancer by 60% [129].

## 5. Shared Preventive Strategies

With the increasing prevalence of obesity, MetS, and D.M., strategies to prevent these metabolic risk factors are urgently needed. Current evidence indicates that the best way to prevent metabolic syndrome and other metabolic risk factors is to adopt a heart-healthy lifestyle, including eating a healthy diet, having enough physical activity, and smoking cessation [109]. The “2013 American Heart Association/American College of Cardiology Guidelines for the Management of Overweight and Obesity in Adults” endorses that lifestyle changes that produce even modest, sustained weight loss of 3–5% produce clinically meaningful health benefits in overweight/obese people with cardiovascular risk factors (high blood pressure, hyperlipidemia, and hyperglycemia) and greater weight losses produce greater benefits [130]. As mentioned before, the beneficial effect of weight loss on the incidence of MI, stroke, and cancer has been demonstrated [130]. Hence, overweight/obese individuals should be prescribed high-intensity (≥14 sessions in 6 months) comprehensive weight loss interventions provided in individual or group sessions by trained health professionals [130].

It has been shown that metabolic risk factors, CVD, and cancer have interrelated pathophysiologic pathways. These shared pathways can offer possible mechanism-based targets for the joint pharmacologic prevention and control of CVDs and cancers [3, 6]. For example, metformin, which is an antiglycaemic drug, has been shown to be associated with an overall 30% lower risk of cancer than other antidiabetic medications, and this protective effect was more prominent in hepatocellular and colorectal cancers [131].

## 6. Role of Individual and the Public Health System in Shared Prevention of CVD and Cancer

Implementing a set of cost-effective “best buy” interventions for CVD and cancer prevention at population and individual levels in health systems is the main role of the public health system to prevent CVD and cancer [132]. These best buy interventions included policies that target populations and service delivery. Evidence revealed that fiscal and regulatory policies to improve shared behavioural and environmental risk factors of CVD and cancer have cost-effective and beneficial effects on the risk of these diseases. Adding a set of services, including vaccination campaigns, screening programs, and lifestyle modifications, can prevent, early detect, and provide treatment before life-threatening adverse events [132, 133]. Integrating CVD and cancer or NCD prevention and care into the existing platforms in each community, including the public health center (PHC) system in LMIC and also relevant private sectors and multinational companies, can address these diseases in proper ways [134].

Effective approaches to reducing the burden of CVD include interventions at the population level and the individual level. The former translated to measures aiming at reducing overall risk factor exposures and the latter to measures aiming at modifying risk factors for high-risk populations. The WHO has suggested a set of crucial measurements for NCD prevention. These guidelines provide protocols and resources for managing NCDs in primary healthcare settings [134]. The WHO-PEN emphasize the importance of intensive behavioural counseling for individuals with a high risk of CVD and consistent lifestyle counseling for everyone. However, systematic lifestyle counseling is not commonly employed as a standard tool for managing high-risk individuals in numerous low- and middle-income countries (LMICs) [134]. Additionally, a considerable number of healthcare professionals lack sufficient training to offer effective counseling, resulting in their advice being often limited to general statements, such as “quit smoking,” “eat healthier,” or “increase physical activity.” Rarely are specific recommendations or referrals provided in such cases. For instance, despite the well-documented effectiveness and cost-effectiveness of brief tobacco interventions, more than half of primary care providers, particularly in LMICs, do not deliver these interventions routinely. A lack of knowledge and skill in tobacco cessation counseling has been proposed as major obstacles in this regard [134]. The HEARTS package [135] improves the application of the WHO-PEN protocols by offering the technical and operational frameworks and tools necessary for integrating CVD management into primary health care [135]. The role of individuals in the prevention of CVD and cancer could be summarized in “self-care” strategies. These strategies include staying fit and healthy and avoiding hazardous behavioural habits such as smoking. Counseling patients on self-care could be integrated into existing care structures.

## 7. Cardio-Oncology Rehabilitation

Advances in the early detection and management of cancer patients have significantly improved their disease-free survival. Many of these patients are at a significantly increased risk of mortality from noncancer causes, particularly CVD. Hence, in addition to the clear need for joint prevention of CVD and cancer in disease-free subjects, effective strategies are needed to mitigate the CVD risk in cancer patients. One of the viable strategies in this regard is the cardio-oncology rehabilitation program that identifies patients at a high risk of CVD, including cardiotoxicity related to cancer therapies, and uses the multimodality approach of cardiac rehabilitation (e.g., exercise plus nutritional counseling and controlling and reducing shared risk factors) to prevent or mitigate cardiovascular events [136, 137].

## 8. Conclusion

An effective joint preventive strategy for CVD and cancer includes a mixture of policies and population-wide interventions to reduce overall risk factor exposure and individual approaches or self-care strategies to modify risk factors for a high-risk population. There are multiple shared lifestyle-related risk factors for CVD and cancer, and controlling them could play a major role in decreasing the burden of both diseases. Smoking cessation and avoiding opium abuse, limiting alcohol consumption, promoting a healthy diet, sufficient physical activity, and healthy sleep, reducing exposure to air, water, and soil pollution, and preventing and treating obesity and metabolic syndrome are among joint preventive strategies for both CVD and cancer. Although there are clear widely accepted targets and programs for improving the status of the majority of the abovementioned risk factors, there is still a lack of appropriate targets and programs for promoting healthy sleep and reducing exposure to environmental pollution.

## Figures and Tables

**Table 1:** WHO targets and strategies for modifiable risk factors for noncommunicablediseases^*∗*^.

Category	WHO target by 2025	Prevention strategies
Physical inactivity	10% reduction in prevalence of insufficient physical activity	(i) Access to safe environments for walking, cycling, and other forms of physical activity
		(ii) Sustainable transport policies
		(iii) National physical activity policies
		(iv) Facilitating active transport to work (e.g., cycling and walking)
		(v) Provision of opportunities and programs for physical activity in schools

Unhealthy diet	30% relative reduction in mean intake of sodium	(i) Identifying sodium rich foods
		(ii) Setting national policies for sodium reduction

Tobacco use	At least 30% reduction in the global prevalence of tobacco	(i) Rising taxes on tobacco
		(ii) Banning smoking in indoor public places
		(iii) Warning people of the dangers of tobacco
		(iv) Banning all forms of tobacco advertising
		(v) Offering help to quite tobacco use

Excess alcohol consumption	>10% reduction in harmful use of alcohol	(i) Raising taxes on alcohol
		(ii) Restricting alcohol advertising
		(iii) Regulating public availability
		(iv) Drunk driving policies
		(v) Counseling for alcohol withdrawal

^*∗*^Global status report on noncommunicable diseases 2014. Geneva, 2014.

**Table 2:** Public health strategies to improve behavioural and environmental risk factors of cardiovascular diseases and cancer.

Category	Public health strategies
Physical inactivity	(i) Counseling about appropriate physical activity in every session by health professional

Unhealthy diet	(i) Promoting public awareness about healthy diet
	(ii) Offering counseling in primary care [23]
	(iii) Emphasising WHO best buys on reducing dietary intake of sodium, sugar, and fat

Tobacco use	(i) Advising by physician in every visiting session
	(ii) Assistance individual, group, or telephone counseling about quitting
	(iii) Behavioural cessation therapies by training in problem solving
	(iv) Treatments with more person-to-person contact and intensity
	(v) Pharmacotherapy (e.g., nicotine replacement or other drugs such as bupropion and varenicline) [11]
	(vi) Psychosocial support enhances motivation
	(vii) Enforcing WHO Framework Convention on Tobacco Control (FCTC)

Opium abuse	(i) Improving data collection to guide resource allocation
	(ii) Increasing safer prescribing
	(iii) Stigma-reduction campaigns
	(iv) Increased spending on harm reduction and treatment [117]

Excess alcohol consumption	(i) Monitoring alcohol consumption by health professionals in their patient
	(ii) Providing brief interventions
	(iii) Counseling and pharmacotherapy

Air pollution	(i) Staying indoors
	(ii) Reducing outdoor air infiltration to indoors
	(iii) Cleaning indoor air with air filters
	(iv) Limiting physical exertion, especially outdoors and near air pollution sources
	(v) Avoiding exposure to air pollutants, especially important for susceptible individuals with chronic cardiovascular diseases
	(vi) Increasing residential green spaces were lacking

**Table 3:** The recommended levels of air pollutants by the WHO air quality guidelines^*∗*^.

Pollutants	Levels
PM_2.5_	10 *μ*g/m^3^ annual mean
	25 *μ*g/m^3^24-hour mean

PM_10_	20 *μ*g/m^3^ annual mean
	50 *μ*g/m^3^24-hour mean
Ozone (O_3_)	100 *μ*g/m^3^8-hour mean

Nitrogen dioxide (NO_2_)	40 *μ*g/m^3^ annual mean
	200 *μ*g/m^3^1-hour mean

Sulfur dioxide (SO_2_)	20 *μ*g/m^3^24-hour mean
	500 *μ*g/m^3^ 10-minute mean

^*∗*^Air quality guidelines, global update 2005, World Health Organization, Geneva, 2005.

## Data Availability

No data were used to support this study.
